# New Insights into the Mechanism of Action of PirAB from Vibrio Parahaemolyticus

**DOI:** 10.3390/toxins14040243

**Published:** 2022-03-30

**Authors:** Sonia A. Soto-Rodriguez, Rodolfo Lozano-Olvera, Gabriela Ramos-Clamont Montfort, Edgar Zenteno, José Luis Sánchez-Salgado, Norberto Vibanco-Pérez, Karla G. Aguilar Rendón

**Affiliations:** 1 Laboratorio de Bacteriología, Centro de Investigación en Alimentación y Desarrollo, A.C. Unidad de Acuacultura y Manejo Ambiental, Av. Sábalo-Cerritos S/N A.P. 711, Mazatlán 82112, Sinaloa, Mexico; rlozano@ciad.mx (R.L.-O.); karla.aguilar@ciad.mx (K.G.A.R.); 2 Centro de Investigación en Alimentación y Desarrollo A.C., Carretera Gustavo Enrique Astiazarán Rosas, No. 46, Col. La Victoria, Hermosillo 83304, Sonora, Mexico; gramos@ciad.mx; 3 Departamento de Bioquímica, Facultad de Medicina, Universidad Nacional Autónoma de México, Circuito Exterior, Ciudad Universitaria, Coyoacan, Mexico City 04510, Mexico, Mexico; ezenteno@unam.mx (E.Z.); sanchez@bq.unam.mx (J.L.S.-S.); 4 Laboratorio de Investigación en Biología Molecular e Inmunología, Unidad Académica de Ciencias Químico Biológicas y Farmacéuticas, Universidad Autónoma de Nayarit, Ciudad de la Cultura, Tepic 63190, Nayarit, Mexico; nvivanco@uan.edu.mx

**Keywords:** PirAB, *Vibrio parahaemolyticus*, AHPND, shrimp, microbiota change

## Abstract

PirAB toxins secreted by *Vibrio parahaemolyticus* (*Vp*) harbor the pVA1 virulence plasmid, which causes acute hepatopancreatic necrosis disease (AHPND), an emerging disease in Penaeid shrimp that can cause 70–100% mortality and that has resulted in great economic losses since its first appearance. The cytotoxic effect of PirAB^Vp^ on the epithelial cells of the shrimp hepatopancreas (Hp) has been extensively documented. New insights into the biological role of the PirB^Vp^ subunit show that it has lectin-like activity and recognizes mucin-like O-glycosidic structures in the shrimp Hp. The search for toxin receptors can lead to a better understanding of the infection mechanisms of the pathogen and the prevention of the host disease by blocking toxin–receptor interactions using a mimetic antagonist. There is also evidence that *Vp* AHPND changes the community structure of the microbiota in the surrounding water, resulting in a significant reduction of several bacterial taxa, especially *Neptuniibacter* spp. Considering these findings, the PirAB^vp^ toxin could exhibit a dual role of damaging the shrimp Hp while killing the surrounding bacteria.

## 1. Introduction

In 2013, *Vibrio parahaemolyticus* (*Vp*) strains were first reported as the causal agents of acute hepatopancreatic necrosis disease (AHPND) [1], a highly infectious and emerging enteric disease threatening farmed shrimp that has been recognized by the World Organization for Animal Health (OIE) [2]. This lethal disease of marine shrimp emerged in China in 2009 and was originally known as early mortality syndrome (EMS). This devastating disease has caused great economic losses in the shrimp industries of Asian countries [3,4,5], Mexico [6], South America [7], and the United States [8]. Between 2009 and 2016, China, Thailand, and Mexico reported losses of USD 11 billion, USD 8 billion, and nearly USD 1 billion, respectively, due to AHPND [9].

The virulence of *Vp* strains *(Vp* AHPND) is due to a conjugative plasmid of approximately 70 kbp (pVA1) that expresses a binary PirAB toxin that is homologous to the Pir toxin secreted by *Photorhabdus* spp.; the PirAB toxin is responsible for the characteristic lesions in the shrimp hepatopancreas (Hp) [1,10,11]. To date, multiple AHPND-causing *Vibrio* spp. have been identified from affected shrimp, including *Vibrio harveyi*, *V. owensii, V. campbellii*, and *V. punensis* [4,12,13,14,15,16,17]. AHPND can be caused by strains of several *Vibrio* spp. because the toxin genes *pirAB*^Vp^ reside in a conjugative plasmid, allowing for horizontal transfer between bacterial species [18,19,20]. AHPND is a disease caused by toxigenic bacteria that produce the PirAB toxin; however, as the disease progresses, secondary bacterial colonization of the damaged Hp occurs. The disease represents a special threat to Penaeid shrimp culture due to its diverse etiology, complexity, and rapid pathogenesis, in addition to the widespread nature of this disease. Moreover, the mechanisms of AHPND toxigenesis are not yet understood.

In this review, we present an overview of AHPND, including the disease-associated signs, progression of histopathological lesions, virulence of *Vp* strains, current knowledge of the virulent pVA1 plasmid, changes in bacterial community structure caused by *Vp* AHPND, and possible factors that could induce or inhibit toxin production. Finally, we present new research on putative membrane receptors and potential inhibitors of the PirAB^Vp^ toxin.

## 2. Acute Hepatopancreatic Necrosis Disease in Penaeid Shrimp

To date, AHPND continues to be the bacterial disease of greatest economic importance that affects both tiger shrimp (*Penaeus monodon*) and the Pacific white shrimp (*Penaeus vannamei*) [21]. Although the Hp of decapod crustacea has been the target organ of AHPND, the Australian red claw crayfish (*Cherax quadricarinatus*) were not susceptible to *Vp* AHPND in cohabitation bioassays [22]. The authors hypothesized that there are differences in the putative receptor binding sites between Penaeid shrimp and crayfish, making the PirAB binary toxin unable to bind to the Hp epithelium of the crayfish receptor(s) and thus unable to cause intoxication. 

Shrimp are more susceptible in their early life stages to intoxication by AHPND strains, with a threshold infective density of >10^4^ CFU mL^−1^ [11], and increased mortality of *P. vannamei* inoculated with *Vp* AHPND has been observed at high salinity levels. AHPND is characterized by severe dysfunction of the shrimp Hp accompanied by clinical signs and particular histopathological changes in the acute disease stage [23]. AHPND-affected shrimp exhibit expanded chromatophores, lethargy, anorexia, empty digestive tracts, and pale-to-white Hp color. However, these clinical signs are also commonly observed in other bacterial diseases, such as necrotizing hepatopancreatitis (NHP-B) and septic hepatopancreatic necrosis (SHPN) [24]. Hence, confirmatory diagnosis of AHPND in shrimp should also include the histopathological lesions observed in the acute stage of the disease and the molecular detection of *pirA*^Vp^ and *pirB*^Vp^ genes coupled with bioassays. Diagnosis of AHPND based only the detection of the *pirA^Vp^* and *pirB^Vp^* genes may be inadequate due to the instability of these genes, as observed in strains isolated from different geographical regions [25]. 

### 2.1. Degree of Virulence

The term virulence is used to describe the relative ability of a microorganism to cause disease in a susceptible host and is better known as the degree of pathogenicity [26]. This ability allows for virulence to be quantitatively evaluated. To this end, some mechanisms of pathogenicity have been explored, such as the evasion of host defense mechanisms, antibiotic resistance, lesion severity, percentage of induced death, invasiveness, and toxigenic capacity. Bacterial strains also possess different degrees of virulence [27,28], which may be due to phenotypic or genotypic variations. For example, the genome of a pathogenic *Listeria monocytogenes* strain owes its virulence to its large quantity of anti-sense RNA, which differs from what is observed in non-pathogenic *L. monocytogenes* strains [29].

The expression of *pirA*^Vp^ and *pirB*^Vp^ genes can influence the degree of virulence, with *Vp* AHPND strains of moderate virulence showing low *pirA* gene expression (<0.4 relative expression) and less virulent strains showing high gene expression (2.1-fold relative expression) [5]. Given that *pirA*^Vp^ and *pirB*^V*p*^ genes are located in the same operon in the virulent plasmid (pVA1) [30], theoretically, both genes must be expressed constitutively and thus show similar virulence. However, it is evident that other factors are also involved. The copy number of pVA1 is related to bacterial density [31]. The PirA and PirB proteins (or the PirAB^Vp^ complex) can be differentially secreted by bacterial cells, affecting their virulence in shrimp [3,5,32], or they can be affected by post-translational modifications. A wider band of PirA and PirB proteins from more virulent strains was observed via western blot [33]. However, to date, the role of pVA1, along with the roles of the secreted toxins, in the virulence of bacterial AHPND strains remains unclear.

Lastly, the *Vp* AHPND strains show different lifestyles during experimental infections that could influence pathogenesis. For example, the more virulent *Vp* M0904 preferentially adheres to the bottom surface of the experimental units, whereas the less virulent *Vp* M0607 strain adheres to the bottom and remains suspended in the water column [34]. In Mexico, during the shrimp mortality events associated with *Vp* AHPND, several strains were isolated with different degrees of virulence (in terms of time of death) [11]. In experimental infections under similar laboratory conditions (i.e., immersion assays with *P. vannamei* challenged at a bacterial density of 10^6^ CFU mL^−1^ in natural or synthetic seawater between 8 and 35 g L^−1^), shrimp either reached 100% mortality from 17 h post-inoculation (p.i.) to 72 h p.i. or did not reach this mortality throughout the entire experiment (Table 1). Our observations with Mexican strains indicate that shrimp size is relevant during experimental infections. Small shrimp are more susceptible to AHPND than larger shrimp, which may indicate that the toxicity of PirAB is dose dependent.

As Table 1 shows, some studies did not include the histopathological analysis of infected shrimp to confirm disease development. Moreover, in some cases, histological observations did not indicate AHPND presence or did not correspond to the acute stage of AHPND lesions. The next section describes the progression of histopathological lesions during AHPND.

### 2.2. Histopathology of AHPND

AHPND has usually been described by histopathological analyses [1,3,6,8,11,15,17,23]. Although there are currently several methods for detecting AHPND (clinical signs, histopathology, and molecular techniques), histopathological analysis remains the primary method to confirm positive cases [2] and evaluate the cytotoxic effects of the PirAB toxin [32]. AHPND causes clinical signs like lethargy, erratic swimming, empty gut, discoloration, and Hp atrophy as well as progressive tissue changes that include the massive sloughing of epithelial cells of hepatopancreatic tubules [1].

**Figure 1:**
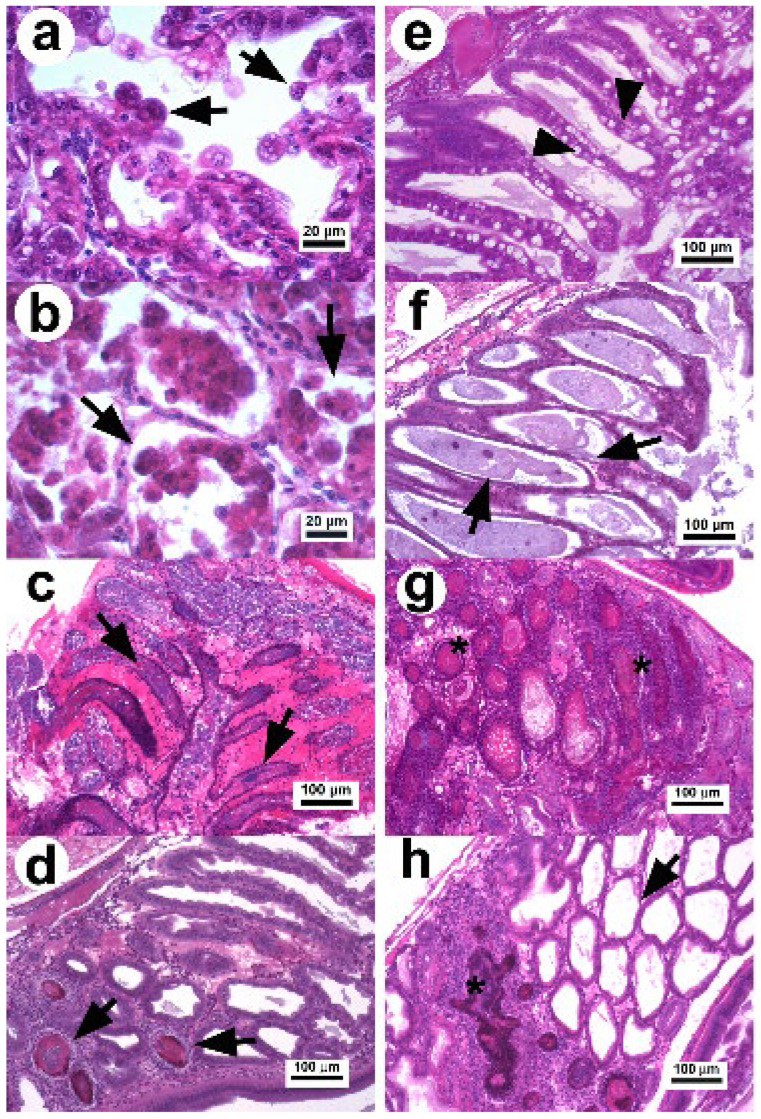
Photomicrograph of the hepatopancreas of *P. vannamei* affected with AHPND. (**a**) Hepatopancreatic tubules in the initial stage: the tubular epithelium undergoes reduction of vacuoles and elongated cells in the lumen (arrow) without evidence of pathogenic bacteria [23]. (**b**) Tissue in the acute stage: the tubular epithelium show massive sloughing of epithelial cells inside the tubular lumen (arrow) [11]. (**c**) Tubules in the terminal stage with hemocytic infiltration in the intertubular tissue, necrotic epithelium, and dead cells with bacterial masses in the tubular lumen (arrow) [23]. (**d**,**e**) Hp in the remission stage with a declination of the necrotic lesions and presence of secondary infection confined (arrow) by melanized hemocytic nodules (**d**,**e**) normal tubular epithelium of recovered shrimp with abundant vacuoles in R and B cells (arrow head) [34]. (**f**) Hepatopancreatic tubules with chronic effect as atrophied epithelium, absence of vacuoles in R cells (arrow), and no evidence of bacteria. (**g**) Tubules with necrotic lesions similar to septic hepatopancreatic necrosis (*). (**h**) Hepatopancreatic tissue with a combined lesion that includes melanized hemocytic nodule lesions associated with septic hepatopancreatic necrosis and tubules with atrophied epithelium associated with a chronic effect. H&E stain.

Histologically, three stages (initial, acute, and terminal) have been commonly reported as part of the pathogenic course of AHPND (Figure 1) [1,6,8,11,23]. However, because of the capacity of shrimp to tolerate the effects of the PirAB toxin, a remission stage of the disease has also been observed in surviving shrimp with E (embryonic) cells acting as bioindicators for this stage [34]. In the initial stage of AHPND, there is a decrease in the number of vacuoles in R and B cells and an elongation of the epithelial cells, which signal cell sloughing (Figure 1a) in the absence of pathogenic bacteria [11,23]. In the acute stage, a massive sloughing of epithelial cells (R, F, and B) occurs, which accumulate in the lumen of the affected tubules (Figure 1b). This is the most remarkable histopathological aspect used for the clinical diagnosis of AHPND; furthermore, mitotic activity in the E cells is absent [1,11,40].

In the terminal stage, the epithelium of hepatopancreatic tubules is entirely necrotic, dead cells appear in different degrees of lysis within the lumen, and there is a proliferation of bacteria associated with the necrotic material, indicating a secondary infection (Figure 1c) [1,11,23,40]. The inflammatory response increases over time causing melanization and hemocytic nodules and capsules around the affected tubules, which delimit disease progression.

In 2020, two studies [34,41] found that surviving shrimp (3 d p.i. with *Vp* AHPND) were able to develop larger melanized necrotic lesions similar to those of septic hepatopancreatic necrosis (SHPN). Thus, surviving shrimp can decrease the cytotoxic effect caused by the PirAB^Vp^ toxin and enter into a remission stage of the disease. This stage is characterized by the reactivation of the mitotic activity in E cells, a decrease in the clinical signs associated with AHPND, and reduced mortality [34]. In addition, the necrotic lesions associated with the terminal stage of AHPND quickly decrease over the p.i. time period (Figure 1d). The histopathological evidence suggests that under experimental conditions, the development of AHPND lesions can follow three main routes from the terminal stage to disease recovery [34]. Surviving shrimp after 5 d p.i. show different histopathological conditions [34,41], which include recovered shrimp displaying normal Hp structures without evidence of lesions (Figure 1e), shrimp with atrophied hepatopancreatic epitheliums without vacuoles in R cells associated with chronic effects (Figure 1f), shrimp with lesions similar to those of SHPN (Figure 1g) and the persistence of a secondary bacterial infection that is delimited by hemocytic nodules, and shrimp displaying a combination of chronic effects and SHPN-associated lesions (Figure 1h).

The development of lesions in AHPND-affected shrimp is associated with the bacterial density, degree of strain virulence [3,11,23,35], PirA and PirB toxin concentrations [32], and infection time [34]. It has been observed that the delay in development time of acute-stage AHPND lesions in *P. vannamei* was dependent on the degree of strain virulence [34]. The acute stage occurred in the first 4 h p.i. with the most virulent strain, whereas this time was 8 h later with the less virulent strain. However, the terminal stage was present at 24 h p.i. for both strains and remained until 48 h p.i. with only the most virulent strain. Shrimp displayed a marked immune response, including hemocytic infiltration, hemocytic nodules, and melanization, to delimit bacterial proliferation and the damage caused by PirAB toxins [34]. The development and persistence of the terminal stage seems to depend on the degree of damage, genetic line, and immunological status of the shrimp.

It has also been previously reported that a minimum concentration of 10 µg g^−1^ of PirA and PirB toxin is necessary to induce the typical lesions of AHPND [32]. Toxin concentrations below 5 µg g^−1^ do not cause the disease, although that concentration can induce the collapse (atrophy) of the tubular hepatopancreatic epithelium [32]. Atrophied epitheliums have also been observed under experimental conditions in shrimp infected with *Vp* AHPND doses lower than the infective threshold (<10^4^ CFU mL^−1^) [11] in low virulence strains [3,11,36,39] and in surviving shrimp [34]. Under conditions of experimental infection, the atrophied epithelium in surviving shrimp could be the result of a decrease in PirAB production/secretion after the acute stage of AHPND, which reduces the lesions caused by the disease and favors shrimp survival. Likewise, Aguilar-Rendón et al. [34] suggest that the atrophied epithelium might be due to the combined effects of continuous exposure to low concentrations of the PirAB toxin. Further studies should be conducted to quantify toxin production of *Vp* AHPND and understand its dynamics during in vivo experiments.

## 3. Virulence Plasmid pVa1

AHPND is mainly caused by *Vp*, which harbors a plasmid of ~70 kbp (pVA1) containing the *pirA*^Vp^ and *pirB*^Vp^ genes that encode the delta-endotoxin responsible for the typical lesions in shrimp Hp [1,10]. The genomes of pVA1-harboring *Vp* revealed a large pan genome with high genetic diversity grouped into three main clades and specific structural differences, in addition to the instability of the *pirAB*^Vp^ region of the pVA1 plasmid [25]. The structural differences found in pVA1 are likely due to the horizontal propagation of the plasmid to other *Vibrio* species [19], such as *V. harveyi* [4,18], *V. campbellii* [18], *V. owensii* [18], and *V. punensis* [17]. These processes might result in the appearance of new pathogenic AHPND strains, which would pose a major threat to the shrimp industry. Likewise, this variability in structural elements could eventually influence their niche adaptation ability, growth behavior, and virulence/pathogenesis.

**Figure 2:**
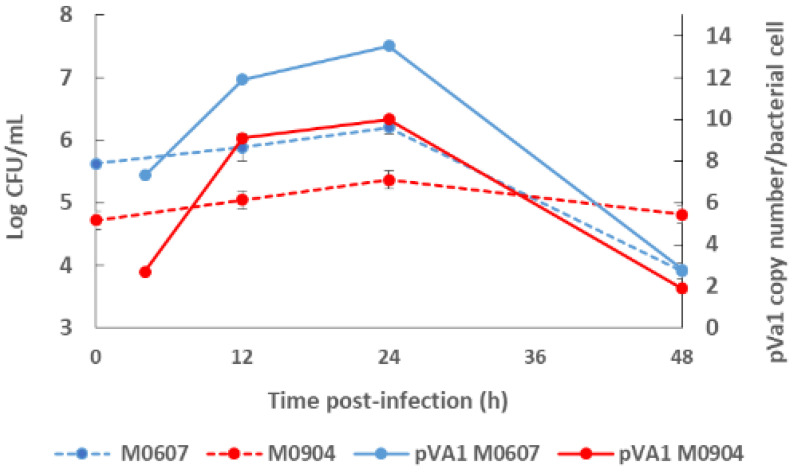
Bacterial density of the bottom seawater and virulent plasmid copy (pVA1) number of moderate virulence *Vibrio parahaemolyticus* strain M0607 and high virulent *V. parahaemolyticus* strain M0904 during experimental infections at 10^5^ CFU mL^−1^.

Recently, Aguilar-Rendón et al. [34] found large variability of the plasmid copy number (7 to 121 copies) per bacterial cell of *Vp* AHPND strains analyzed by qPCR [30,42], although it has been reported that virulence does not depend on the copy number of *pirA*^Vp^/*pirB*^Vp^ genes [33]. To date, no clear evidence of their role in AHPND has been found. A study using a shotgun metagenomics approach on bottom seawater with *P. vannamei* inoculated with two *Vp* AHPND strains registered more than one copy of pVA1 per bacterial cell (1.9 to 13.5 copies per bacterial cell) throughout the experimental infection period [31]. In this study, the copy number of the virulent plasmid was not dependent on the degree of virulence of the *Vp* AHPND strain but rather on bacterial density (Figure 2). Nonetheless, few studies have evaluated the variability in the plasmid copy number per bacterial cell in relation to the degree of virulence or bacterial density and how this may influence AHPND pathogenesis.

## 4. Changes in the Microbiota of Seawater

Much remains to be understood of the microbial communities present in the seawater of cultured shrimp [43,44]. Most studies have focused on bacterial community structures of the intestinal microbiota [45,46,47] or the effects of environmental factors on the microbial community in shrimp farms [48]. Proteobacteria dominate the gut microbiota of Penaeid shrimp, and the microbiome is involved in the regulation of shrimp health and disease [49]. Most studies of *P. vannamei* affected with AHPND have focused on characterizations of the bacterial communities of the Hp, stomach, intestines, or sediment using 16S rRNA amplicons [50,51,52]. Toxins affect the microbial communities of their host [53], yet few studies have evaluated the surrounding microbiota of the seawater in the presence of diseased aquatic organisms [54]. The type III secretion system (T3SS) has been suggested to be the mechanism by which the PirAB^Vp^ toxin is secreted [32]. However, the type VI secretion system (T6SS) is involved in the virulence of human pathogenic *Vp* strains through the secretion of effector proteins, which are toxic to surrounding bacteria [55]. In addition, T6SS1 of a *Vp* AHPND isolate was functional during the challenge of *P. vannamei* [56].

Recently, the changes in the water microbiome of juvenile *P. vannamei* inoculated with moderately virulent and highly virulent *Vp* strains (M0607 and M0904, respectively) were studied using the shotgun metagenomics sequencing approach [31]. In this study, the Proteobacteria phylum was found to be dominant in the water, according to the bacterial community associated with AHPND [57]. At the family level, *Rhodobacteraceae* was the most predominant taxon, which has already been detected in the microbiota of both healthy and diseased shrimp and in the culture water [44,50,57]. The abundance of *Oceanospirillaceae* appears to be related to environmental conditions [44], and *Vibrionaceae* are notable during AHPND infection [51,57].

*Neptuniibacter* spp. are common in seawater and associated with farmed organisms [58]. The dominance of the Neptuniibacter complex shows high genetic variation in the initial community structure [31]. A significant and marked reduction was observed in the reads assigned to *Neptuniibacter* spp. after inoculation with M0607 and M0904, particularly with *Vp* M0904 from 4 h p.i. onward. A gradual increase at 48 h of *Pseudoalteromonas stutzeri*, *Halomonas* sp., and *Marinobacter adhaerens* was also observed.

The depletion pattern in the Neptuniibacter complex suggests that these species could be highly affected by the bacterial toxins secreted from both *Vp* strains [59], particularly the PirB^Vp^ subunit given its lectin-like activity [60]. The reduction in abundance of the Neptuniibacter complex suggests that bacterial competition could be mediated by T6SS, which regulates bacterial interactions [59,61]. Some *Vp* AHPND strains contain active T3SS1, T6SS1, and T6SS2 [62]. Aguilar-Rendón et al. [31] observed an enrichment of the functions associated with these systems that was related to inoculation with *Vp* strains, and these functions were closely associated with bacterial pathogenesis [63]. T6SS represents complex secretion machinery and contributes to competitive survival or pathogenesis in many Gram-negative bacteria [56]. Three effector proteins of T6SS were only detected in inoculated treatments (primarily in the M0904 strain): (a) cytotoxin Hcp; (b) the temperature-dependent protein that activates T6SS according to environmental conditions [59]; and (c) the antitoxin serine/threonine protein kinase [31], which is a type of immunity protein that protects the bacterial community against self-intoxication due to effector proteins from T6SS [64]. The T6SS1 system is active under specific temperature (30 °C) and salinity (3% NaCl) conditions, which were maintained during experimental infections [59], and thus it could be functional in both *Vp* AHPND strains. This antibacterial system, which is found in 12 strains of *Vp* AHPND, mediates interspecific and intraspecific competition, promoting shrimp infection [62]. It is strongly suggested that both *Vp* AHPND strains could employ T6SS1 as a selective advantage during shrimp intoxication by killing surrounding bacteria.

## 5. Factors That Could Induce or Inhibit Toxin Production

### 5.1. Quorum Sensing

Quorum sensing (QS) is a cell-to-cell signaling mechanism in response to an increased bacterial cell population [65]. Bacterial QS produce, release, and recognize molecular autoinducers (AIs) that bind to surface bacterial receptors, triggering signal transduction cascades that alter the expression of genes related with survival and infection factors, such as sporulation, luminescence, biofilm formation, and virulence [66]. The QS mechanism is widely distributed in *Vibrionaceae* members, with the acyl-homoserine lactones (AHLs) being among the more common AIs. For example, AHLs have been implicated in the signaling mechanisms that activate the production of luciferase in *V. fischeri* [65]. In addition, *V. harveyi* produces and responds to three other AIs: (1) HAI-1, [N-(3- hydroxy butyryl)-homoserine lactone], an intra-species AI; (2) CAI-1, [(Z)-3-aminoundec-2- en-4one], which is restricted to the *Vibrio* genera; and (3) the inter-species AI-2 [(2S,4S)-2-methyl-2,3,3,4-tetrahydroxytetrahydrofuran-borate]. These three AIs act in parallel to regulate over 600 target genes through complex signaling cascades [65,67]. The capacity of *Vibrionaceae* for “sensing self” and “sensing others” allows for both competition and cooperation in complex microbial communities [68].

Virulence gene expression regulated by QS has been studied extensively in *V. harveyi* and may serve as a basis for understanding the QS mechanisms in *Vp* given that this pathogen contains the central conserved components of the QS pathway known in *V. harveyi* [69]. For example, a LuxT homolog of *V. harveyi*, SwrT, activates genes that encode for translocation across surfaces and swarming and is lateral-flagella-driven in *Vp* [70,71]. In addition, *V. harveyi* and presumably *Vp* produce three types of AIs, namely auto inducer 2 (AI-2), harveyi auto inducer 1 (HAI1), and cholerae auto inducer 1 (CAI1), which are recognized by the surface membrane receptors LuxP/LuxQ, LuxN, and CqsS, respectively [72]. In a preliminary study, [73] showed that the production of PirAB^Vp^ binary toxin is regulated by the AI-2 QS process. They tested the effect of a cell-free supernatant from *V. harveyi* containing AI-2 (CFS-VH) on an AHPND-causing *Vp* strain. The AI-2-containing supernatant accelerated the production time and yield of both PirA^Vp^ and PirB^Vp^ toxins, whereas the application of the furanone [(5Z)-4-bromo5-(bromomethylene)-2(5H)-furanone] AI-2 antagonist delayed AHPND toxin production or secretion. This study opens new perspectives on QS mechanisms in *Vp* and on possible treatments and management strategies to control AHPND infection in shrimp culture. Interestingly, AI-2 is synthetized by numerous bacterial species and can facilitate inter-species cell–cell signaling [74], resulting in changes of *Vp* behavior in complex microbial communities.

### 5.2. Environmental Factors

Bacterial adaptation and survival depend on the capacity to properly respond to changes in internal and external environments. The survival of *Vibrio* spp. in marine environments depends on carbon and energy sources, dissolved oxygen, water pH, salinity, temperature, and starvation [75]. In particular, changes in temperature due to global warming are a growing concern for aquaculture due to the increased risk of *Vp*-induced diseases. Environmental stress can increase horizontal gene transfer mechanisms in AHPND-causing *Vp* strains, promoting their growth [76,77] and increasing the risk of AHPND outbreaks and disease dispersion in tropical zones. Recently, the effect of temperature shifts on *pirA*^Vp^ and *pirB*^Vp^ gene expression of the AHPND-*Vp* AAHMRU04 strain isolated from white shrimp exhibiting clinical signs of AHPND was evaluated [78]. Bacteria were grown at 30 °C for 24 h and subsequently exposed to a set of different temperature trials for 4 days. The *pirA*^Vp^ and *pirB*^Vp^ genes were induced when the temperature shifted from high (26–32 °C) to low (22–28 °C) [78].

The relationship between salinity and AHPND in *P. vannamei* was studied by [79]. Pathogen-free shrimp cultures (5, 10, 15, and 20 g L^−1^ of NaCl) were challenged with a *Vp* AHPND broth. In all salinity treatments, *Vp* AHPND caused infection in shrimp as confirmed by histological damage and the presence of *pirAB*^Vp^ toxin genes by PCR analysis. However, cumulative mortality was different, showing higher survival in shrimp maintained at lower salinities. Since *Vp* reproduces more efficiently in high salinity environments, it is likely that a greater amount of PirAB^Vp^ toxin was produced, resulting in a higher cumulative mortality in *P. vannamei* when maintained under these conditions. However, different patterns were observed when challenging *P. vannamei* growing under different salinity conditions with the *Vp* AHPND strain E9 [80]. In this study, mortality was higher at lower salinities and a positive correlation was present with the expression of the *pirA*^Vp^ gene. Although more experiments are needed to determine the influence of salinity on the expression of *pirAB*^Vp^, these experiments corroborate that the toxin can be expressed at different salinities [81] and that the management of salinity in shrimp culture can be an important factor to control *Vp* infectivity.

Another environmental factor that has been studied with regard to the production of the PirAB^vp^ binary toxin is related to fluid shear and the hydrodynamic forces acting on *Vp* due to either natural influences or the use of aquaculture equipment to enhance shrimp productivity, such as blowers or aerators [82]. To this end, the effect of shaking conditions on the AHPND-causing *Vp* M0904 was studied [83]. At a constant agitation of 110 rpm, bacteria developed cellular aggregates together with levan (branched polymeric fructans)-containing biofilm formations and acquired tolerance against antimicrobial agents (kanamycin, ampicillin, rifampicin, and tetracycline), possibly due to high biofilm production. In addition, a significant decrease was observed not only in PirA^Vp^/PirB^Vp^ toxin production but also in the virulence of *Vp* M0904 to *Artemia* and *Macrobrachium* larvae. Increasing the shaking speed to 120 rpm produced an increase in PirA^Vp^/PirB^Vp^ toxin production, the virulence of *Vp* M0904 to *Artemia* and *Macrobrachium* larvae, and the expression of polar flagellin (flaA), polar flagellin-specific chaperone (fliS), and chemotaxis protein (CheR). This type of study provides valuable information for understanding the behavior of *Vp* AHPND in aquaculture environments [83].

### 5.3. Biofilm Formation

The formation of bacterial biofilms represents one of the most important survival mechanisms, attachment, as well as host colonization strategies of bacteria [84]. This phenomenon is influenced by abiotic and biotic factors regulated by QS [85]. ToxR is an important virulence regulator implicated in the synthesis of *Vp* biofilms that also controls the expression of the virulence factors found in human pathogenic *Vp,* including thermostable direct hemolysin (TDH), TDH-related hemolysin (TRH), and T3SS [86,87]. The expression of these factors is regulated by QS through the production of and responses to AI-2 [85,88,89]. Under these conditions, biofilm and toxin production appear to be simultaneous activities.

Information on the relationship between biofilm formation and the production of PirAB^vp^ binary toxin in *Vp* AHPND is lacking. The only study to address this issue is that of [83], which observed an inverse relationship between the production of biofilms and that of the PirAB^Vp^ toxin. This behavior refers to the formation of abiotic films in response to fluid shear and hydrodynamic forces. However, the regulation, growth kinetics, and characteristics of *Vp* AHPND biofilms in the host and their relationships with PirAB^vp^ toxin production remain uncharacterized.

## 6. Search for Membrane Receptors of PirA^Vp^ and PirB^Vp^

### 6.1. Biological Activities of the PirA^Vp^ and PirB^Vp^ Subunits

Bacterial protein toxins, like PirAB^Vp^, are molecular self-governing virulence factors that target specific host cells, triggering different damaging processes involved in the disease of the infected organism. The binding of bacterial toxins to plasma cell surface receptors is an essential first step for shrimp intoxication. Knowing the structures of these receptors can further the understanding of the infection mechanisms with the aim of preventing host disease by blocking the toxin–receptor interaction using a mimetic antagonist [90]. The PirA^Vp^/PirB^Vp^ toxin induces cell damage in the shrimp Hp, although it is not seen in other organs, and is considered a shrimp-specific toxin [91]. Moreover, it seems that PirA^Vp^/PirB^Vp^ receptors will be found exclusively in this organ [92]. Recently, it has been observed that the B Subunit of the PirAB^Vp^ toxin is an amino sugar-specific lectin-like, and it is able to recognize glycoproteins on the epithelium of the Hp, suggesting its participation in AHPND pathogenesis [60,93]. Nevertheless, the PirA^Vp^/PirB^Vp^ binding model complex requires clarification and further information is needed.

It is known that PirA^Vp^ and PirB^Vp^ form a heterodimeric complex that binds to receptors located on the cells of the shrimp Hp [92,94]. However, the precise nature of the toxin receptors is still not known. Lee et al. [10] suggested that PirAB^Vp^ structure is homologous to the *insecticidal Photorhabdus* insect-related (Pir) binary *toxin,* and in silico analysis showed that the PirA^Vp^ and PirB^Vp^ toxins possess similar structures to the functional domains of the pore-forming *Bacillus thuringiensis* Cry toxins [10,92]. The structural alignment of both toxins indicates that the PirA^Vp^ subunit is similar to the lectin-like recognition domain III of *B. huringiensis* toxin, whereas PirB^Vp^ corresponds to the pore-forming I and II domains [10,92,95,96]. In this context, the initial interaction of the PirA^Vp^/PirB^Vp^ toxins would be through lectin-carbohydrate recognition between PirA^Vp^ and the glycans exposed on the surface of the plasma membrane of Hp cells [92]. Structural features and molecular docking of the PirA^Vp^ subunit show a potential sugar-binding cavity for glycans containing the *N*-Acetylgalactosamine (GalNac) molecule, whereas the PirB^Vp^ subunit structure contains a C-terminal receptor domain similar to Cry domain II for protein–protein ligand interactions and an N-terminal consistent with other membrane pore-forming toxins, including Cry domain I [10,95]. In addition, Hao et al. [91] analyzed the distribution and homology of PirAB^Vp^-like proteins in other bacterial species and showed that at least seven bacterial taxa harbor complete or partial *pir*AB genes, including *Alcaligenes, Photorhabdus, Pectobacterium carotovorum, Vibrio, Xenorhabdus*, *Yersinia*, and *Shewanella violacea*. All examined PirB proteins examined by Hao et al. [91] showed typical *B. thuringiensis* Cry structure formed by several α-helix bundles in the N-terminal of PirB and a coup of parallel or anti-parallel β-sheets in the C-terminal of PirB^Vp^.

**Figure 3:**
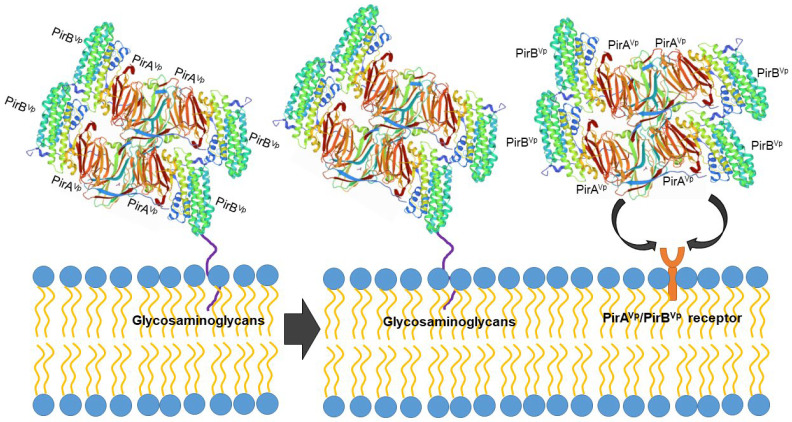
Proposed PirAB^Vp^ binding scheme. The PirA^Vp^/PirB^Vp^ heterotetrametric complex first uses PirB^Vp^-lectin to recognize and bind with glycosaminoglycan molecules; meanwhile, PirA^Vp^ stabilizes the complex. Then the complex probably binds to the receptor molecules on the membrane of the hepatopancreatic epithelial cells of shrimp to trigger the massive sloughing of these cells.

However, the protein structure in the receptor binding sites of compared PirB proposed by Lin et al. [92] reflected an evolutionary divergence in the amino acid sequences (for more details, see Hao et al. [91]). The conformation and the direction of Loop 2 of PirB are unique in *V. parahaemolyticus*, thus PirB^Vp^ might target a specific receptor in the cell membrane. In addition, the predicted structures of PirA toxins also showed remarkable differences in ligand-binding sites. These structural variations could largely influence the recognition events of PirAB^Vp^. We propose that PirAB^Vp^ forms a heterotetrametric complex containing four PirA^Vp^ subunits and four PirB^Vp^ subunits [92] and that PirB^Vp^ first recognizes glycosaminoglycan molecules as mucin-like or beta-hexosaminidase where the Gal(β1–3/1–4)GlcNAc(α1–2) sequence is essential for PirB^Vp^ recognition in the hepatopancreatic membrane [93] (Figure 3). The role of the PirA subunit might be stabilizing the complex for a better binding to the possible receptor molecule on the shrimp hepatopancreatic epithelial cells [92]. A complete understanding of the receptor binding mechanisms of PirA/PirB toxins is essential in order to elucidate the toxin mechanism.

Recent studies have suggested that the regions of interaction of PirAB^Vp^ are different than those of insecticidal toxins. From the extracellular products (ECPs) of *Vp*, a heterotetrametric complex of 250 kDa has been purified, which contains four PirA^Vp^ and four PirB^Vp^ subunits. The PirB^Vp^ subunit was confirmed to show lectin-like activity and the recognition of mucin-like O-glycosidic structures in the shrimp Hp that may act as receptors for toxin binding, while PirA^Vp^ did not present this activity [60]. Lectin activity has been suggested due to its ability to interact specifically with oligosaccharides and glycoproteins such as mucin, but further structural assays will confirm the participation of the lectin effect in the pathogenesis of *Vp* AHPND.

The PirAB^Vp^ complex seems to be necessary to induce AHPND signs. The mechanism of action of the entire toxin during the AHPND disease process remains to be determined. However, experiments conducted with the recombinant proteins rPirA^Vp^ and rPirB^Vp^ showed that only the PirAB^Vp^ complex and rPirB^Vp^ displayed Mg^2+^ or Ca^2+^ independent hemagglutinating activity (HA) toward rat red cells, whereas rPirA^Vp^ was not able to agglutinate erythrocytes from several animal species [60].

In a first attempt to determine the sugar specificity of the putative PirB^Vp^ lectin-like, subsequent competition experiments were conducted using a wide battery of monosaccharides, disaccharides, and glycoproteins. D-galactosamine (GalNH2) and N-unsubstituted glucosamine (GlcNH_2_) monosaccharides were better sugar inhibitors for rPirB^Vp^ than any of the other tested monosaccharides or disaccharides. Among glycoconjugates, the fetuin glycoprotein showed the strongest rPirB^Vp^ HA inhibition capacity, whereas egg white chicken ovalbumin and heparin showed relative inhibitory potency. With this in mind, the PirB^Vp^ subunit binds to a glycoconjugate glycan moiety containing amino sugars [60]. Further experiments conducted by the same group showed the existence of different glycan receptors for PirB^Vp^, and in particular a mucin-like receptor located at the surface membrane of the cell Hp and an internal hexosaminidase glycoprotein receptor that is possibly involved in toxin-related cell damage to shrimp tissues [93]. Beta-hexosaminidase (β-*N*-acetyl hexosaminidase) is a ubiquitous lysosomal enzyme with multiple roles in protein glycosylation and synthesis and glycoconjugate metabolism [97]. This glycoprotein plays an important role in arthropod molting and chitin degradation and in the defense system of *P. vannamei* against parasites [98,99]. Extracellular beta-hexosaminidases secreted by eukaryotes occur as dimers and possess N-glycosidically-linked glycans with oligomannosidic and complex-type glycan structures [100,101]. The possibility that PirB^Vp^ could recognize N-linked oligosaccharides expressed by endosomal or secreted beta-hexosaminidase, which would allow for an increased pathogenesis of *Vp* in crustaceans, cannot be excluded.

Previous data suggest putative lectin-like PirB^Vp^ subunit activity [60,93] that contrasts with the functions of domains I and II proposed for the Cry toxin and with the proposed function of the PirA^Vp^ subunit given that it has not been possible to verify that this subunit can recognize carbohydrates in the experiments conducted to date. In light of this, the PirA^Vp^ subunit could play an initial stabilizing role, allowing PirB^Vp^ to bind with higher affinity to the glycan receptors located at the surface of Hp cells.

### 6.2. Expression of Mucin-like O-Glycosidic Structures in Shrimp

O-glycans are critical for the development and proper functioning of multicellular organisms. Mucin-type glycans are widely found on the cell surfaces and secreted glycoconjugates of invertebrates [102]. These O-glycans may serve as receptor-binding sites for a variety of pathogenic bacteria and their toxins [103]. A small unit of *P. vannamei* hemocyanin had O-glycans that were closely associated with agglutination activity toward *Vibrio fluvialis, V*. *alginolyticus,* and *V. parahaemolyticus* [104,105].

A mucin-like peritrophin-like gene from fleshy shrimp (*Fenneropenaeus chinensis)* is able to bind to Gram-negative bacteria [106], while another mucin-like peritrophin-like gene from the shrimp *Exopalaemon carinicauda* is involved in white spot syndrome viral infections [107]. In addition, a mucin-like peritrophin has been implicated in *V. harveyi* infection in the black tiger shrimp *P. monodon* [108]. Abiotic characteristics, such as decreases in temperature and changes in diet, increase the expression of several mucin-like proteins in *P. vannamei* [109,110,111]. These modifications could be related to the pathologic development of *Vibrio* infection, increasing the number of binding targets in the shrimp digestive system. Searching for possible receptors for the lectin-like PirB^Vp^ [93] has yielded evidence of some correspondence with a mucin-like protein expressed in the shrimp Hp of *P. vannamei*. These studies are the beginning of a better understanding of the infection mechanisms of *Vp* in shrimp.

### 6.3. Receptor on Shrimp Hemocytes

The PirAB^Vp^ toxin is known to mainly target the epithelial cells of shrimp Hp tubules. In addition, [112] found that the PirAB^Vp^ toxin binds to the epithelial cells of the digestive tract and produces similar lesions in the midgut and hindgut regions in germ-free brine shrimp *Artemia*. Moreover, the dysregulation of apoptosis-related genes in *Vp* AHPND-challenged *P. vannamei* hemocytes suggests that *Vp* AHPND induces apoptosis in hemocytes [113]. For the *B.* *thuringiensis* Cry toxin, apoptosis is induced by a series of processes that start with the interaction between the Cry1A toxin and carbohydrate moiety (surface receptor binding) of an N aminopeptidase (APN) [114].

In the transcriptome of *Vp* AHPND-challenged *P*. *vannamei*, an aminopeptidases N1 (*LvAPN1*) gene was identified [115]. DNA sequence analysis of the *LvAPN1* gene showed a putative C-terminal transmembrane domain and various putative N- and O-glycosylation sites. The expression of *LvAPN1* increases in hemocytes after challenging *P*. *vannamei* with either *Vp* AHPND or the partially purified *Vp* AHPND toxins. Silencing of *LvAPN1* significantly reduced *LvAPN1* transcription levels in the stomach, Hp, and hemocytes and increased the survival of adult *P*. *vannamei* that were challenged with the partially purified *Vp* AHPND toxins. These observations suggest the putative role of *LvAPN1* as a PirAB^Vp^ toxin receptor located on the hemocyte surface [115].

Other putative carbohydrate receptors for the PirAB^Vp^ toxin could be located in the surface of *P. vannamei* hemocytes, as these cells express a plethora of glycoconjugates. Using commercial lectins with different carbohydrate specificities, the presence of carbohydrate moieties containing mainly N-acetyl-glucosamine (GlcNAc) and N-acetylneuraminic acid (sialic acid) was demonstrated [116]. In another study, these carbohydrates were recognized by the rPirB^Vp^ subunit [60].

## 7. Search of Potential Inhibitors of the PirABVp Toxin

Understanding the structural biology of PirAB^Vp^ is essential for finding or developing antiadhesive agents or receptor analogs that could prevent adhesion and subsequent cell entry of the toxin, thus inhibiting its activity. In particular, it is important to decipher the roles and structural features of complex carbohydrates that serve as toxin receptors. According to research by our group, the PirB subunit presents lectin-like activity, and its adhesion can be inhibited in the presence of fucosylated glycans and by those that contain N-acetyl glucosamine [60,93].

In addition to glycans, studies of peptides that can interact with PirAB^Vp^ are also needed. Computational tools like molecular docking can play an important role in the search for antiadhesive peptides or in the design of antiadhesive peptide analogs through the creation of precise structural models of peptide-toxin complexes and the calculation of binding free energies [117,118]. The search for bifunctional peptides that can be used to improve shrimp growth while at the same time protecting them from the PirAB^Vp^ toxin is also important. For example, oilseed peptides have been found to contribute to improved shrimp health and growth performance when used as feed ingredients [119]. In silico studies have revealed six dual-target peptides from different oilseed proteins capable of interfering with the formation of the PirA^Vp^/PirB^Vp^ complex. Such peptides (1139–2977 Da in mass and 10–28 residues in length) are possible candidates for the future development of peptide-based anti-AHPND agents [118].

## 8. Concluding Remarks and Future Perspectives

To date, the potential mechanisms of PirAB^Vp^ that cause AHPND in Penaeid shrimp remain unknown. Although much is currently known about the pVA1 virulent plasmid, there is a lack of information regarding the variability of the plasmid copy number per bacterial cell and how this influences the pathogenesis of AHPND. It has been suggested that *Vp* AHPND strains could use T6SS1 as a selective advantage during shrimp intoxication by killing the surrounding bacteria. Future studies on the types and activities of effector proteins of T6SS in *Vp* AHPND during infection could facilitate the development of strategies to control AHPND-causing strains. Although studies have reported that environmental factors like salinity can affect the production of toxins and shrimp survival, more experiments are needed to determine the influence of salinity on the expression of *pirAB*^Vp^ genes and the pathogenesis of AHPND. Furthermore, there is evidence that the PirA^Vp^ subunit could play an initial stabilizing role, allowing PirB^Vp^ to bind with higher affinity to different glycan receptors, such as the mucin-like receptor located at the surface of the cellular membranes of the Hp and an internal hexosaminidase glycoprotein receptor that could be involved in the PirAB^Vp^ toxin. These studies are the beginning of a better understanding of the infection mechanisms of *Vp* in shrimp.

Understanding the structural biology of PirAB^Vp^ is essential for finding or developing antiadhesive agents or receptor analogs that could prevent adhesion and the subsequent entry of the toxin into the cell. It is also important to elucidate the roles and structural features of complex carbohydrates that serve as toxin receptors. The PirB^Vp^ subunit presents lectin-like activity, and thus its adhesion can be inhibited by glycans. In this sense, research with putative glycomimetic antagonists like fucoidan will provide new directions for the future development of PirAB^Vp^ inhibitors.

## Figures and Tables

**Table 1:** Pathogenicity of *Vibrio* species responsible for AHPND in Penaeid shrimp.

Strain	Origin	Shrimp Size (g)	Density (CFU/mL)	Histo.	First Dead-100% Mortality (h)	Reference
Vp 13-028A/3	Vietnam	0.5–2.0	2 × 10^6^	Yes	<24–48	[1]
Vp 3HP	Thailand	~2.0	1 × 10^6^	Yes	ND-24	[3]
Vp S02	China	~2.0	1 × 10^6^	Yes	ND-24	[3]
Vp 13-306D/4	Mexico	~2.0	ND	Yes	>24–72	[6]
Vp 13-511A/1	Mexico	~3.0	2 × 10^6^	Yes	ND–24	[6]
Vp M0607	Mexico	0.5–1.0	7.8 × 10^6^	Yes	15–48 *	[11]
Vp M0802	Mexico	0.5–1.0	3.3 × 10^6^	Yes	7–25	[11]
Vp M0904	Mexico	0.5–1.0	2.2 × 10^6^	Yes	4–17	[11]
Vp 2S01	China	~ 1.0	1 × 10^6^	Yes	3–18	[16]
Vp-BA94C2	Latin America	2.5 ± 0.5	2 × 10^6^	Yes	6–70	[17]
Vp D6	Thailand	3 5	1 × 10^6^	ND	144–216	[33]
Vp D6	Thailand	0.82	5 × 10^5^	ND	24–96	[33]
Vp GD10	China	~2.0	~×10^6^	Yes	<24–72	[35]
Vp 5HP	Thailand	1.8 ± 0.2	~×10^6^	Yes	>24–96 *	[36]
Vp XN89	Vietnam	1.8 ± 0.2	~×10^6^	Yes	>24–96 *	[36]
Vp 15-250/20	Latin America	1–1.5	2 × 10^6^	Yes	<12–168 *	[37]
Vp 19-021-D1	Korea	1–1.5	2 × 10^6^	Yes	<12–168 *	[37]
Vp 19-022-A1	Korea	1–1.5	2 × 10^6^	Yes	<12–168 *	[37]
Vp C3	Thailand	2.0	2 × 10^5^	Yes	ND-72	[38]
Vpu-BA55	Latin America	2.5 ± 0.5	2 × 10^6^	Yes	8–70 *	[17]
Vc 20130629003S01	China	~1.0	2 ×10^6^	Yes	12–36	[16]
Vc 16-904/1	Latin America	2.0	2 × 10^5^	Yes	ND-72	[38]
Vc 20130629003S01	China	~1.0	1 × 10^6^	Yes	3–24	[15]
Vc 34	Peru	1.2	~×10^6^	Yes	<24–120	[39]
Vc 36	Peru	1.2	~×10^6^	Not	<24–120	[39]
Vc 43	Peru	1.2	~×10^6^	Not	<24–120	[39]
Vo SH-14	China	0.5–2.0	~×10^6^	Yes	12–96	[13]
Vo SH-14	China	0.5–2.0	~×10^6^	ND	<20–40 *	[18]

Vp: *Vibrio parahaemolyticus*; Vpu: *Vibrio punensis*; Vc: *Vibrio campbellii*; Vo: *Vibrio owensii*; * shrimp did not reach 100% cumulative mortality; Histo: Histopathology study; Yes: Typical histopathological lesions of AHPND acute stage; ND: not determinate; Not: No histopathological lesions of AHPND acute stage.

## Data Availability

The study did not report any data.

## References

[1] Tran L.H., Nunan L., Redman R.M., Mohney L.L., Pantoja C.R., Fitzsimmons K., Lightner D.V. (2013). Determination of the infectious nature of the agent of acute hepatopancreatic necrosis syndrome affecting Penaeid shrimp. Dis. Aquat. Org..

[2] OIE (2019). Manual of Diagnostic Tests for Aquatic Animals (2019).

[3] Joshi J., Srisala J., Truong V.H., Chen I.T., Nuangsaeng B., Suthienkul O., Lo C.F., Flegel T.W., Sritunyalucksana K., Thitamadee S. (2014). Variation in *Vibrio parahaemolyticus* isolates from a single Thai shrimp farm experiencing an outbreak of acute hepatopancreatic necrosis disease (AHPND). Aquaculture.

[4] Kondo H., Van P.T., Dang L.T., Hirono I. (2015). Draft genome sequence of non-*Vibrio parahaemolyticus* acute hepatopancreatic necrosis disease strain KC13.17.5, isolated from diseased shrimp in Vietnam. Genome Announc..

[5] Feng B., Liu H., Wang M., Sun X., Pan Y. (2017). Diversity analysis of acute hepatopancreatic necrosis disease-positive *Vibrio parahaemolyticus* strains. Aquac. Fish..

[6] Nunan L., Lightner D., Pantoja C., Gomez-Jimenez S. (2014). Detection of acute hepatopancreatic necrosis disease (AHPND) in Mexico. Dis. Aquat. Org..

[7] Restrepo L., Bayot B., Betancourt I., Pinzón A. (2016). Draft genome sequence of pathogenic bacteria *Vibrio parahaemolyticus* strain Ba94C2, associated with acute hepatopancreatic necrosis disease isolate from South America. Genom. Data.

[8] Dhar A.K., Piamsomboon P., Caro L.F.A., Kanrar S., Adami R., Juan Y.S. (2019). First report of acute hepatopancreatic necrosis disease (AHPND) occurring in the USA. Dis. Aquat. Org..

[9] Shinn A.P., Pratoomyot J., Griffiths D., Trong T.Q., Vu N.T., Jiravanichpaisal J., Briggs M. (2018). Asian shrimp production and the economic costs of disease. Asian Fish. Sci..

[10] Lee C.T., Chen I.T., Yang Y.T., Ko T.P., Huang Y.T., Huang J.Y., Huang M.F., Lin S.J., Chen C.Y., Lin S.S. (2015). The opportunistic marine pathogen *Vibrio parahaemolyticus* becomes virulent by acquiring a plasmid that expresses a deadly toxin. Proc. Natl. Acad. Sci. USA.

[11] Soto-Rodriguez S.A., Gomez-Gil B., Lozano-Olvera R., Betancourt-Lozano M., Morales-Covarrubias M.S. (2015). Field and experimental evidence of *Vibrio parahaemolyticus* as the causative agent of acute hepatopancreatic necrosis disease (AHPND) of cultured shrimp (*Litopenaeus vannamei*) in northwestern Mexico. Appl. Environ. Microbiol..

[12] Liu L., Xiao J., Xia X., Pan Y., Yan S., Wang Y. (2015). Draft genome sequence of *Vibrio owensii* strain SH-14, which causes shrimp acute hepatopancreatic necrosis disease. Genome Announc..

[13] Liu L., Xiao J., Zhang M., Zhu W., Xia X., Dai W., Pan Y., Yan S., Wang Y. (2018). A *Vibrio owensii* strain as the causative agent of AHPND in cultured shrimp, *Litopenaeus vannamei*. J. Invertebr. Pathol..

[14] Ahn Y.S., Piamsomboond P., Tang K.F.J., Han J.E., Kim J.H. (2017). Complete genome sequence of AHPND-causing *Vibrio campbellii* LA16-V1 isolated from *Penaeus vannamei* cultured in a Latin American country. Genome Announc..

[15] Dong X., Wang H., Xie G., Zou P., Guo C., Liang Y., Huang J. (2017). An isolate of *Vibrio campbellii* carrying the pirVP gene causes acute hepatopancreatic necrosis disease. Emerg. Microbes Infect..

[16] Dong X., Wang H., Zou P., Chen J., Liu Z., Wang X., Huang J. (2017). Complete genome sequence of *Vibrio campbellii* strain 20130629003S01 isolated from shrimp with acute hepatopancreatic necrosis disease. Gut Pathog..

[17] Restrepo L., Bayot B., Arciniegas S., Bajaña L., Betancourt I., Panchana F., Reyes Muñoz A. (2018). PirVP genes causing AHPND identified in a new *Vibrio* species (*Vibrio punensis*) within the commensal *Orientalis* clade. Sci. Rep..

[18] Xiao J., Liu L., Ke Y., Li X., Liu Y., Pan Y., Yan S., Wang Y. (2017). Shrimp AHPND-causing plasmids encoding the PirAB toxins as mediated by pirAB-Tn903 are prevalent in various *Vibrio* species. Sci. Rep..

[19] Dong X., Chen J., Song J., Wang H., Wang W., Ren Y., Guo C., Wang X., Tang K.F.J., Huang J. (2019). Evidence of the horizontal transfer of pVA1-type plasmid from AHPND-causing *V. campbellii* to non-AHPND *V. owensii*. Aquaculture.

[20] Dong X., Song J., Chen J., Bi D., Wang W., Ren Y., Wang H., Wang G., Tang K.F.J., Wang X. (2019). Conjugative transfer of the pVA1-type plasmid carrying the *pirABvp* genes results in the formation of new AHPND-causing *Vibrio*. Front. Cell. Infect. Microbiol..

[21] FAO (2013). Report of the FAO/MARD Technical Workshop on Early Mortality Syndrome (EMS) or Acute Hepatopancreatic Necrosis Syndrome (AHPNS) of Cultured Shrimp (under TCP/VIE/3304), Hanoi, Vietnam, 25 to 27 June 2013.

[22] Powers Q., Caro L.F.A., Fitzsimmons K.M., McLain J.E., Dhar A.K. (2021). Crayfish (*Cherax quadricarinatus*) susceptibility to acute hepatopancreatic necrosis disease (AHPND). J. Invertebr. Pathol..

[23] Soto-Rodriguez S.A., Gomez-Gil B., Lozano-Olvera R., Bolan-Mejía C., Aguilar-Rendon K.G., Enciso-Ibarra J. (2018). Pathological, genomic and phenotypical characterization of *Vibrio parahaemolyticus*, causative agent of acute hepatopancreatic necrosis disease (AHPND) in Mexico. Asian Fish. Sci..

[24] Lightner D.V. (1996). A Handbook of Shrimp Pathology and Diagnostic Procedures for Diseases of Cultured Penaeid Shrimp.

[25] González-Gómez J.P., Soto-Rodriguez S., López-Cuevas O., Castro-del Campo N., Chaidez C., Gomez-Gil B. (2020). Phylogenomic Analysis Supports Two Possible Origins for Latin American Strains of *Vibrio parahaemolyticus* Associated with Acute Hepatopancreatic Necrosis Disease (AHPND). Curr. Microbiol..

[26] Salyer A., Whitt D. (2002). Bacterial Pathogenesis, a Molecular Approach.

[27] Donnenberg M.S. (2000). Pathogenic strategies of enteric bacteria. Nature.

[28] Casadevall A., Pirofski L. (2001). Host-pathogen interactions: The attributes of virulence. J. Infect. Dis..

[29] Wurtzel O., Sesto N., Mellin J.R., Karunker I., Edelheit S., Bécavin C., Archambaud C., Cossart P., Sorek R. (2012). Comparative transcriptomics of pathogenic and non-pathogenic *Listeria* species. Mol. Syst. Biol..

[30] Han J.E., Tang K.F.J., Tran L.H., Lightner D.V. (2015). *Photorhabdus* insect-related (Pir) toxin-like genes in a plasmid of *Vibrio parahaemolyticus*, the causative agent of acute hepatopancreatic necrosis disease (AHPND) of shrimp. Dis. Aquat. Org..

[31] Aguilar-Rendón K.G., Soto-Rodriguez S.A., Gomez-Gil B., Lozano-Olvera R., Yáñez-Rivera B. (2022). Water microbiome dynamics of Pacific white shrimp *Penaeus vannamei* infected with *Vibrio parahaemolyticus* strains responsible for acute hepatopancreatic necrosis disease. Aquaculture.

[32] Sirikharin R., Taengchaiyaphum S., Sanguanrut P., Chi T.D., Mavichak R., Proespraiwong P., Nuangsaeng B., Thitamadee S., Flegel T.W., Sritunyalucksana K. (2015). Characterization and PCR detection of binary, Pir-Like toxins from *Vibrio parahaemolyticus* isolates that cause acute hepatopancreatic necrosis disease (AHPND) in shrimp. PLoS ONE.

[33] Tinwongger S., Nochiri Y., Thawonsuwan J., Nozaki R., Kondo H., Awasthi S.P., Hinenoya A., Yamasaki S., Hirono I. (2016). Virulence of acute hepatopancreatic necrosis disease PirAB-like relies on secreted proteins not on gene copy number. J. Appl. Microbiol..

[34] Aguilar-Rendón K.G., Lozano-Olvera R., Yáñez-Rivera B., Soto-Rodriguez S.A. (2020). Bacteriological and histopathological analysis of *Penaeus vannamei* experimentally infected with *Vibrio parahaemolyticus*-AHPND strains. Dis. Aquat. Org..

[35] Hong X.P., Xu D., Zhuo Y., Liu H.Q., Lu L.Q. (2016). Identification and pathogenicity of *Vibrio parahaemolyticus* isolates and immune responses of *Penaeus* (*Litopenaeus*) *vannamei* (Boone). J. Fish Dis..

[36] Phiwsaiya K., Charoensapsri W., Taengphu S., Dong H.T., Sangsuriya P., Nguyen G.T.T., Pham H.Q., Amparyup P., Sritunyalucksana K., Taengchaiyaphum S. (2017). A natural *Vibrio parahaemolyticus* ΔpirAVppirBVp+ mutant kills shrimp but produces neither PirVp toxins nor acute hepatopancreatic necrosis disease lesions. Appl. Environ. Microbiol..

[37] Han J.E., Choi S.K., Han S.H., Lee S.C., Jeon H.J., Lee C., Lee K.J. (2020). Genomic and histopathological characteristics of *Vibrio parahaemolyticus* isolated from an acute hepatopancreatic necrosis disease outbreak in Pacific white shrimp (*Penaeus vannamei*) cultured in Korea. Aquaculture.

[38] Han J.E., Tang K.F.J., Aranguren L.F., Piamsomboon P. (2017). Characterization and pathogenicity of acute hepatopancreatic necrosis disease natural mutants, pirAB_vp_(-) *Vibrio parahaemolyticus*, and pirAB_vp_ (+) *Vibrio campbellii* strains. Aquaculture.

[39] Vicente A., Taengphu S., Hung A.L., Mora C.M., Dong H.T., Senapin S. (2020). Detection of *Vibrio campbellii* and *V. parahaemolyticus* carrying full-length pirABVp but only *V. campbellii* produces PirVp toxins. Aquaculture.

[40] Lightner D.V., Redman R.M., Pantoja C.R., Noble B.L., Tran L.H. (2012). Early mortality syndrome affects shrimp in Asia. Glob. Aquac. Advocate.

[41] Caro L.F.A., Mai H.N., Noble B., Dhar A.K. (2020). Acute hepatopancreatic necrosis disease (*VP*_AHPND_), a chronic disease in shrimp (*Penaeus vannamei*) population raised in Latin America. J. Invertebr. Pathol..

[42] Han J.E., Tang K.F.J., Lightner D.V. (2015). Genotyping of virulence plasmid from *Vibrio parahaemolyticus* isolates causing acute hepatopancreatic necrosis disease in shrimp. Dis. Aquat. Org..

[43] Cardona E., Gueguen Y., Magré K., Lorgeoux B., Piquemal D., Pierrat F., Noguier F., Saulnier D. (2016). Bacterial community characterization of water and intestine of the shrimp *Litopenaeus stylirostris* in a biofloc system. BMC Microbiol..

[44] Md Zoqratt M., Eng W.W.H., Thai B.T., Austin C.M., Gan H.M. (2018). Microbiome analysis of Pacific white shrimp gut and rearing water from Malaysia and Vietnam: Implications for aquaculture research and management. PeerJ.

[45] Rungrassamee W., Klanchui A., Chaiyapechara S., Maibunkaew S., Tangphatsornruang S., Jiravanichpaisal P., Karoonuthaisiri N. (2013). Bacterial population in intestines of the black tiger shrimp (*Penaeus monodon*) under different growth stages. PLoS ONE.

[46] Zhang M., Sun Y., Chen K., Yu N., Zhou Z., Chen L., Du Z., Li E. (2014). Characterization of the intestinal microbiota in Pacific white shrimp, *Litopenaeus vannamei*, fed diets with different lipid sources. Aquaculture.

[47] Tzeng T.D., Pao Y.Y., Chen P., Weng F., Jean W.D., Wang D. (2015). Effects of host phylogeny and habitats on gut microbiomes of oriental river prawn (*Macrobrachium nipponense*). PLoS ONE.

[48] Zhang H., Sun Z., Liu B., Xuan Y., Jiang M., Pan Y., Zhang Y. (2016). Dynamic changes of microbial communities in *Litopenaeus vannamei* cultures and the effects of environmental factors. Aquaculture.

[49] Holt C.C., Bass D., Stentiford G.D., van der Giezen M. (2021). Understanding the role of the shrimp gut microbiome in health and disease. J. Invertebr. Pathol..

[50] Chen W.Y., Ng T.H., Wu J.H., Chen J.W., Wang H.C. (2017). Microbiome dynamics in a shrimp grow-out pond with possible outbreak of acute hepatopancreatic necrosis disease. Sci. Rep..

[51] Yao Z., Yang K., Huang L., Huang X., Qiuqian L., Wang K., Zhang D. (2018). Disease outbreak accompanies the dispersive structure of shrimp gut bacterial community with a simple core microbiota. AMB Express.

[52] Yang Q., Dong X., Xie G., Fu S., Zou P., Sun J., Wang Y., Huang J. (2019). Comparative genomic analysis unravels the transmission pattern and intra-species divergence of acute hepatopancreatic necrosis disease (AHPND)-causing *Vibrio parahaemolyticus* strains. Mol. Genet. Genom..

[53] Kohl K.D., Dearing M.D. (2016). The woodrat gut microbiota as an experimental system for understanding microbial metabolism of dietary toxins. Front. Microbiol..

[54] Xiong J., Zhu J., Dai W., Dong C., Qiu Q., Li C. (2017). Integrating gut microbiota immaturity and disease discriminatory taxa to diagnose the initiation and severity of shrimp disease. Environ. Microbiol..

[55] Yu Y., Yang H., Li J., Zhang P., Wu B., Zhu B., Zhang Y., Fang W. (2012). Putative type VI secretion systems of *Vibrio parahaemolyticus* contribute to adhesion to cultured cell monolayers. Arch. Microbiol..

[56] Lien Y.W., Lai E.M. (2017). Type VI secretion effectors: Methodologies and biology. Front. Cell. Infect. Microbiol..

[57] Pinoargote G., Flores G., Cooper K., Ravishankar S. (2018). Effects on survival and bacterial community composition of the aquaculture water and gastrointestinal tract of shrimp (*Litopenaeus vannamei*) exposed to probiotic treatments after an induced infection of acute hepatopancreatic necrosis disease. Aquaculture.

[58] Diéguez A.L., Balboa S., Magnesen T., Romalde J.L. (2017). Neptuniibacter pectenicola sp. nov. and *Neptuniibacter marinus* sp. nov., two novel species isolated from a Great scallop (*Pecten maximus*) hatchery in Norway and amended description of the genus *Neptuniibacter*. Syst. Appl. Microbiol..

[59] Salomon D., Gonzalez H., Updegraff B.L., Orth K. (2013). *Vibrio parahaemolyticus* Type VI secretion system 1 is activated in marine conditions to target bacteria, and is differentially regulated from system 2. PLoS ONE.

[60] Victorio-De Los Santos M., Vibanco-Pérez N., Soto-Rodriguez S., Pereyra A., Zenteno E., Cano-Sánchez P. (2020). The B Subunit of PirAB(vp) Toxin Secreted from *Vibrio parahaemolyticus* Causing AHPND Is an Amino Sugar Specific Lectin. Pathogens.

[61] Fu S., Wang L., Tian H., Wei D., Liu Y. (2018). Pathogenicity and genomic characterization of *Vibrio parahaemolyticus* strain PB1937 causing shrimp acute hepatopancreatic necrosis disease in China. Ann. Microbiol..

[62] Li P., Kinch L.N., Ray A., Dalia A.B., Cong Q., Nunan L.M., Camilli A., Grishin N.V., Salomon D., Orth K. (2017). Acute hepatopancreatic necrosis disease-causing *Vibrio parahaemolyticus* strains maintain an antibacterial type VI secretion system with versatile effector repertoires. Appl. Environ. Microbiol..

[63] Johnson D.I., Johnson D.I. (2018). Bacterial virulence factors. Bacterial Pathogens and Their Virulence Factors.

[64] Russell A.B., Singh P., Brittnacher M., Bui N.K., Hood R.D., Carl M.A., Agnello D.M., Schwarz S., Goodlett D.R., Vollmer W. (2012). A widespread bacterial type VI secretion effector superfamily identified using a heuristic approach. Cell Host Microbe.

[65] Papenfort K., Bassler B.L. (2016). Quorum sensing signal-response systems in Gram-negative bacteria. Nat. Rev. Microbiol..

[66] Rutherford S.T., Bassler B.L. (2012). Bacterial quorum sensing: Its role in virulence and possibilities for its control. Cold Spring Harb. Perspect. Med..

[67] Federle M.J., Bassler B.L. (2003). Interspecies communication in bacteria. J. Clin. Investig..

[68] Wellington S., Greenberg E.P. (2019). Quorum sensing signal selectivity and the potential for interspecies cross talk. mBio.

[69] Makino K., Oshima K., Kurokawa K., Yokoyama K., Uda T., Tagomori K., Iijima Y., Najima M., Nakano M., Yamashita A. (2003). Genome sequence of *Vibrio parahaemolyticus*: A pathogenic mechanism distinct from that of *V. cholerae*. Lancet.

[70] Jaques S., McCarter L.L. (2006). Three new regulators of swarming in *Vibrio parahaemolyticus*. J. Bacteriol..

[71] Eickhoff M.J., Fei C., Huang X., Bassler B.L. (2021). LuxT controls specific quorum-sensing-regulated behaviors in *Vibrionaceae* spp. via repression of qrr1, encoding a small regulatory RNA. PLoS Genet..

[72] Zhang Y., Qiu Y., Tan Y., Guo Z., Yang R., Zhou D. (2012). Transcriptional regulation of opaR, qrr2-4 and aphA by the master quorum-sensing regulator OpaR in *Vibrio parahaemolyticus*. PLoS ONE.

[73] Pumkaew M., Taengchaiyaphum S., Powtongsook S., Pungrasmi W., Sritunyalucksana K. (2019). Production of acute hepatopancreatic necrosis disease toxin is affected by addition of cell-free supernatant prepared from Al-2-producing *Vibrio harveyi* mutant. J. World Aquac. Soc..

[74] Federle M.J. (2009). Autoinducer-2-based chemical communication in bacteria: Complexities of interspecies signaling. Contrib. Microbiol..

[75] Takemura A.F., Chien D.M., Polz M.F. (2014). Associations and dynamics of *Vibrionaceae* in the environment, from the genus to the population level. Front. Microbiol..

[76] Williams S.L., Jensen R.V., Kuhn D.D., Stevens A.M. (2017). Analyzing the metabolic capabilities of a *Vibrio parahaemolyticus* strain that causes Early Mortality Syndrome in shrimp. Aquaculture.

[77] Fu S., Wei D., Yang Q., Xie G., Pang B., Wang Y., Lan R., Wang Q., Dong X., Zhang X. (2020). Horizontal plasmid transfer promotes the dissemination of Asian acute hepatopancreatic necrosis disease and provides a novel mechanism for genetic exchange and environmental adaptation. mSystems.

[78] Pragthong P., Chirapongsatonkul N. (2020). Temperature-dependent expression of virulence genes in *Vibrio parahaemolyticus* AHPND strain (VpAHPND). Int. J. Agric. Technol..

[79] Schofield P.J., Noble B.L., Caro L.F.A., Mai H.N., Padilla T.J., Millabas J., Dhar A.K. (2021). Pathogenicity of Acute Hepatopancreatic Necrosis Disease (AHPND) on the freshwater prawn, *Macrobrachium rosenbergii*, and Pacific White Shrimp, *Penaeus vannamei*, at various salinities. Aquac. Res..

[80] López-Cervantes G., Álvarez-Ruiz P., Luna-Suárez S., Luna-González A., Esparza-Leal H.M., Castro-Martínez C., Gámez-Jiménez C., Soto-Alcalá J. (2021). Temperature and salinity modulate virulence and PirA gene expression of *Vibrio parahaemolyticus*, the causative agent of AHPND. Aquac. Int..

[81] Soto-Rodriguez S., Lozano Olvera R., Palacios-Gonzalez D., Bolan-Mejía M., Aguilar-Rendon K. (2019). Characterization and growth conditions of *Vibrio parahaemolyticus* strains with different virulence degrees that cause acute hepatopancreatic necrosis disease in *Litopenaeus vannamei*. J. World Aquac. Soc..

[82] Sultana T., Haque M., Salam M., Alam M. (2017). Effect of aeration on growth and production of fish in intensive aquaculture system in earthen ponds. J. Bangladesh Agric. Univ..

[83] Kumar V., Roy S., Baruah K., Van Haver D., Impens F., Bossier P. (2020). Environmental conditions steer phenotypic switching in acute hepatopancreatic necrosis disease-causing *Vibrio parahaemolyticus*, affecting PirAVP/PirBVP toxins production. Environ. Microbiol..

[84] Flemming H.-C., Wuertz S. (2019). Bacteria and archaea on Earth and their abundance in biofilms. Nat. Rev. Microb..

[85] Mizan M.F., Jahid I.K., Kim M., Lee K.H., Kim T.J., Ha S.D. (2016). Variability in biofilm formation correlates with hydrophobicity and quorum sensing among *Vibrio parahaemolyticus* isolates from food contact surfaces and the distribution of the genes involved in biofilm formation. Biofouling.

[86] Zhang Y., Hu L., Osei-Adjei G., Zhang Y., Yang W., Yin Z., Lu R., Sheng X., Yang R., Huang X. (2018). Autoregulation of ToxR and Its Regulatory Actions on Major Virulence Gene Loci in *Vibrio parahaemolyticus*. Front. Cell. Infect. Microbiol..

[87] Yildiz F.H., Visick K.L. (2009). *Vibrio* biofilms: So much the same yet so different. Trends Microbiol..

[88] Henke J.M., Bassler B.L. (2004). Quorum sensing regulates type III secretion in *Vibrio harveyi* and *Vibrio parahaemolyticus*. J. Bacteriol..

[89] Zhu J., Miller M.B., Vance R.E., Dziejman M., Bassler B.L., Mekalanos J.J. (2002). Quorum-sensing regulators control virulence gene expression in *Vibrio cholerae*. Proc. Natl. Acad. Sci. USA.

[90] Sharon N. (2006). Carbohydrates as future anti-adhesion drugs for infectious diseases. Biochim. Biophys. Acta Gen. Subj..

[91] Hao J., Zhang Y., Fu S., Lu Y., Hua X., Liu Y. (2019). Pathogenicity and protein analysis of photorhabdus insect-related (Pir) toxin PirAB revealed PirABvp is a host-specific toxin. Aquaculture.

[92] Lin S.J., Hsu K.C., Wang H.C. (2017). Structural insights into the cytotoxic mechanism of *Vibrio parahaemolyticus* PirAvp and PirBvp toxins. Mar. Drugs.

[93] De Los Santos M.V., Sánchez-Salgado J.L., Pereyra A., Zenteno E., Vibanco-Pérez N., Ramos-Clamont Montfort G., Soto-Rodriguez S.A. (2022). The *Vibrio parahaemolyticus* subunit toxin PirB(vp) recognizes glycoproteins on the epithelium of the *Penaeus vannamei* hepatopancreas. Comp. Biochem. Physiol. B Biochem. Mol. Biol..

[94] Lin S.J., Chen Y.F., Hsu K.C., Chen Y.L., Ko T.P., Lo C.F., Wang H.C., Wang H.C. (2019). Structural insights to the heterotetrameric interaction between the *Vibrio parahaemolyticus* PirAvp and PirBvp toxins and activation of the cry-like pore-forming domain. Toxins.

[95] Sengupta A., Sarkar A., Priya P., Ghosh Dastidar S., Das S. (2013). New insight to structure-function relationship of GalNAc mediated primary interaction between insecticidal Cry1Ac toxin and HaALP receptor of *Helicoverpa armigera*. PLoS ONE.

[96] Kitami M., Kadotani T., Nakanishi K., Atsumi S., Higurashi S., Ishizaka T., Watanabe A., Sato R. (2011). *Bacillus thuringiensis* cry toxins bund specifically to various proteins via domain III, which had a galactose-binding domain-like fold. Biosci. Biotechnol. Biochem..

[97] Fantus I.G., Goldberg H.J., Whiteside C.I. (2006). The Hexosamine Biosynthesis Pathway.

[98] Xie X.-L., Huang Q.-S., Wang Y., Ke C.-H., Chen Q.-X. (2009). Modification and Modificatory Kinetics of the Active Center of Prawn β-N-Acetyl-D-glucosaminidase. J. Biomol. Struct. Dyn..

[99] Song Y., Evenseth L.M., Iguchi T., Tollefsen K.E. (2017). Release of chitobiase as an indicator of potential molting disruption in juvenile *Daphnia magna* exposed to the ecdysone receptor agonist 20-hydroxyecdysone. J. Toxicol. Environ. Health Part A.

[100] Ettrich R., Kopecký V., Hofbauerová K., Baumruk V., Novák P., Pompach P., Man P., Plíhal O., Kutý M., Kulik N. (2007). Structure of the dimeric N-glycosylated form of fungal beta-N-acetyl hexosaminidase revealed by computer modeling, vibrational spectroscopy, and biochemical studies. BMC Struct. Biol..

[101] Weitz G., Proia R.L. (1992). Analysis of the glycosylation and phosphorylation of the alpha-subunit of the lysosomal enzyme, beta- hexosaminidase A, by site-directed mutagenesis. J. Biol. Chem..

[102] Zhu F., Li D., Chen K. (2019). Structures and functions of invertebrate glycosylation. Open Biol..

[103] Erlandson M.A., Toprak U., Hegedus D.D. (2019). Role of the peritrophic matrix in insect-pathogen interactions. J. Insect Physiol..

[104] Zhang Z., Wang F., Chen C., Zheng Z., Aweya J.J., Zhang Y. (2017). Glycosylation of hemocyanin in *Litopenaeus vannamei* is an antibacterial response feature. Immunol. Lett..

[105] Zhang Z., Li R., Aweya J.J., Wang F., Zhong M., Zhang Y. (2019). Identification and characterization of glycosylation sites on *Litopenaeus vannamei* hemocyanin. FEBS Lett..

[106] Du X.-J., Wang J.-X., Liu N., Zhao X.-F., Li F., Xiang J. (2006). Identification and molecular characterization of a peritrophin-like protein from fleshy prawn (*Fenneropenaeus chinensis*). Mol. Immunol..

[107] Wang L., Li F., Xiang J. (2013). A new shrimp peritrophin-like gene from *Exopalaemon carinicauda* involved in white spot syndrome virus (WSSV) infection. Fish Shellfish Immunol..

[108] Soonthornchai W., Rungrassamee W., Karoonuthaisiri N., Jarayabhand P., Klinbunga S., Söderhäll K., Jiravanichpaisal P. (2010). Expression of immune-related genes in the digestive organ of shrimp, *Penaeus monodon*, after an oral infection by *Vibrio harveyi*. Dev. Comp. Immunol..

[109] Duan Y., Wang Y., Liu Q., Dong H., Li H., Xiong D., Zhang J. (2019). Changes in the intestine microbial, digestion and immunity of *Litopenaeus vannamei* in response to dietary resistant starch. Sci. Rep..

[110] Duan Y., Yun W., Ding X., Xiong D., Zhang J. (2019). Response of intestine microbiota, digestion, and immunity in Pacific white shrimp *Litopenaeus vannamei* to dietary succinate. Aquaculture.

[111] Wang Z., Zhou J., Li J., Zou J., Fan L. (2020). The immune defense response of Pacific white shrimp (*Litopenaeus vannamei*) to temperature fluctuation. Fish Shellfish Immunol..

[112] Kumar V., Nguyen D.V., Baruah K., Bossier P. (2019). Probing the mechanism of VPAHPND extracellular proteins toxicity purified from *Vibrio parahaemolyticus* AHPND strain in germ-free *Artemia* test system. Aquaculture.

[113] Zheng Z., Wang F., Aweya J.J., Li R., Yao D., Zhong M., Li S., Zhang Y. (2018). Comparative transcriptomic analysis of shrimp hemocytes in response to acute hepatopancreas necrosis disease (AHPND) causing *Vibrio parahaemolyticus* infection. Fish Shellfish Immunol..

[114] Soberón M., Pardo L., Muñóz-Garay C., Sánchez J., Gómez I., Porta H., Bravo A. (2010). Pore formation by Cry toxins. Adv. Exp. Med. Biol..

[115] Luangtrakul W., Boonchuen P., Jaree P., Kumar R., Wang H.-C., Somboonwiwat K. (2021). Cytotoxicity of *Vibrio parahaemolyticus* AHPND toxin on shrimp hemocytes, a newly identified target tissue, involves binding of toxin to aminopeptidase N1 receptor. PLoS Pathog..

[116] Estrada N., Velázquez E., Rodríguez-Jaramillo C., Ascencio F. (2016). Carbohydrate moieties and cytoenzymatic characterization of hemocytes in white leg shrimp *Litopenaeus vannamei*. Int. J. Cell Biol..

[117] Kuyucak S., Norton R.S. (2014). Computational approaches for designing potent and selective analogs of peptide toxins as novel therapeutics. Future Med. Chem..

[118] Ong J.H., Wong W.L., Wong F.C., Chai T.T. (2021). Targeting PirAvp and PirBvp toxins of *Vibrio parahaemolyticus* with oilseed peptides: An in silico approach. Antibiotics.

[119] Shao J., Zhao W., Han S., Chen Y., Wang B., Wang L. (2019). Partial replacement of fishmeal by fermented soybean meal in diets for juvenile white shrimp (*Litopenaeus vannamei*). Aquac. Nutr..

